# Selective endothelial removal: A case series of a phase I/II surgical trial with long-term follow up

**DOI:** 10.3389/fmed.2022.901187

**Published:** 2022-07-29

**Authors:** Yu-Chi Liu, Yu Qiang Soh, Viridiana Kocaba, Jodhbir S. Mehta

**Affiliations:** ^1^Cornea and Refractive Surgery Group, Singapore Eye Research Institute, Singapore, Singapore; ^2^Tissue Engineering and Cell Therapy Group, Singapore Eye Research Institute, Singapore, Singapore; ^3^Department of Cornea and External Eye Disease, Singapore National Eye Centre, Singapore, Singapore; ^4^Ophthalmology and Visual Sciences Academic Clinical Program, Duke-National University of Singapore (NUS) Medical School, Singapore, Singapore; ^5^Netherlands Institute for Innovative Ocular Surgery, Rotterdam, Netherlands

**Keywords:** Peters anomaly, cornea, endothelium, keratoplasty, regenerative medicine

## Abstract

Peters anomaly is a congenital condition which results in a central corneal opacity from birth. Selective Endothelial Removal (SER) is a novel surgical technique and a form of regenerative therapy, which encourages clearance of the central corneal opacity by the patient’s own corneal endothelial cells, and it may potentially be beneficial for the treatment of Peters anomaly. We have performed a phase I/II surgical trial, evaluating the safety of SER in four eyes (three patients) with Peters Anomaly. These patients underwent SER at between 9 and 39 months of age, each demonstrating clearance of central corneal opacities and improvements in vision post-operatively. No complications occurred in any of these eyes, at a minimal post-operative follow-up duration of 48 months. We conclude that SER for Peters anomaly is a safe surgical procedure. While encouraging efficacy outcomes have been observed, these findings should be further evaluated in a larger scale Phase II/III surgical trial.

## Introduction

Children born with congenital corneal opacities may experience severe visual impairment from birth. Among the wide spectrum of possible etiologies of congenital corneal opacities, Peters anomaly is the most commonly encountered (1). Peters anomaly may be classified into 2 clinical subtypes (2): Type 1 Peters anomaly is characterized by adhesions between the cornea and iris, while type 2 Peters anomaly represents a more severe phenotype with adhesions between the cornea and crystalline lens (2). Both subtypes present clinically as central corneal opacities, which may be associated with other intraocular pathologies including secondary glaucoma, cataracts and posterior segment anomalies. In addition, histopathological studies on Peters anomaly have shown that immature or absent Descemet’s membrane, as well as attenuated endothelial cells, were observed in the area of the corneal opacity (3, 4).

Management for Peters anomaly can be guided by a phenotype-based clinical classification (5). Those with dense, large (>3 mm), centrally located corneal opacities require surgical interventions such as surgical iridectomy (6), full-thickness corneal transplantation (7) and/or lysis of irido-corneal and/or lenticulo-corneal adhesions (i.e., adhesiolysis) (8) to achieve clearance of the central visual axis. However, pediatric penetrating keratoplasty (PK) has been recognized as high-risk procedure because of the difficulties in pre-operative and post-operative assessment, low scleral rigidity and high vitreous pressure during operation, as well as increased fibrin reaction after the surgery (9). The visual outcomes after PK in Peters anomaly remain highly variable. The graft survival was reported as 78% at 1 year (10) and 34% at 10 years post-operatively (7), in addition to other potentially blinding complications such as recurrent graft rejections, infections, secondary glaucoma and retinal detachments (11–15). A recent study including 60 eyes with Peters anomaly further reported the probability of a clear corneal graft at 10 years was 74.2 and 38.9% for type I and type II Peters anomaly, respectively (16). However, we must be mindful that a clear graft does not equate to good vision due to issues with surgical induced astigmatism, anisometropia and amblyopia.

In this article, we describe the results of a case series of a phase I/II trial of a minimally invasive, regenerative therapy-based surgical technique, termed “Selective Endothelial Removal” (SER), which has the potential to enhance the post-operative outcomes of patients with Peters anomaly. This technique was developed following extensive *ex vivo* and *in vivo* animal studies (17, 18) ascertaining key factors promoting endothelial cell regeneration. We performed the first case in 2016 and published the report in 2018 (19). The primary objective of this phase I/II trial was to evaluate the long-term safety of SER for the treatment of Peters anomaly, with the secondary objective being to gain insights into the potential clinical benefits of SER in terms of achievement of post-operative corneal clearance and improvement in visual acuity.

## Materials and methods

**FIGURE 1:**
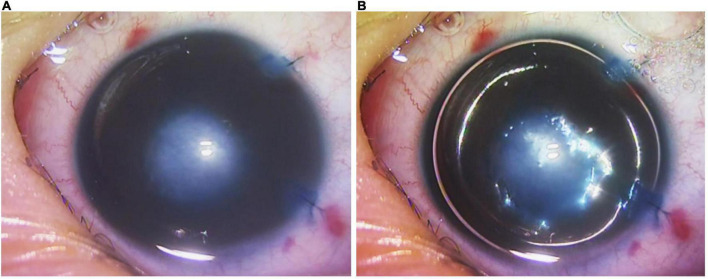
Intra-operative video screenshots of patient 2’s right eye. **(A)** Selective endothelial removal resulted in the intentional creation of a central endothelial defect underlying the central corneal opacity, evidenced by positive staining with trypan blue. **(B)** Following trypan blue staining, a full intracameral air bubble was injected. The absence of a reflective ring around the central region of trypan blue staining indicated that the Descemet’s membrane was intact despite selective endothelial removal.

Selective endothelial removal was performed under general anesthesia, either as an isolated procedure, or in combination with other intraocular surgeries such as cataract extraction, adhesiolysis and vitrectomy. The surgical details have been described before (19). In brief, the surgery was initiated by the insertion of an anterior chamber maintainer via a clear corneal incision, which prevented intra-operative collapse of the anterior chamber with a constant infusion of balanced-salt-solution. Iridocorneal adhesiolysis was performed with a Sinskey hook. A custom made, angulated 30G cannula with soft silicone tip (ASICO, IL; Item AS-7661) was inserted into the anterior chamber through a 1 mm-wide paracentesis at the peripheral cornea. Thereafter it was applied to the posterior aspect of the central corneal opacity with gentle scraping to dislodge the anomalous corneal endothelial cells without damaging the underlying DM. Intraoperative, intracameral trypan blue (VisionBlue, Dutch Ophthalmic) staining followed by air-bubble injection was performed to ascertain the successful removal of corneal endothelial cells (Figure 1A) and post-scraping preservation of DM (Figure 1B), respectively (19). The margins of an endothelial defect showed an absence of a reflective ring on the posterior corneal surface, confirming intact DM after endothelium removal (Supplementary Video 1). All corneal incisions were closed with 10-0 nylon sutures, and the patients were discharged with a tailing course of post-operative steroid and antibiotic eyedrops.

## Results

### Case 1

**FIGURE 2:**
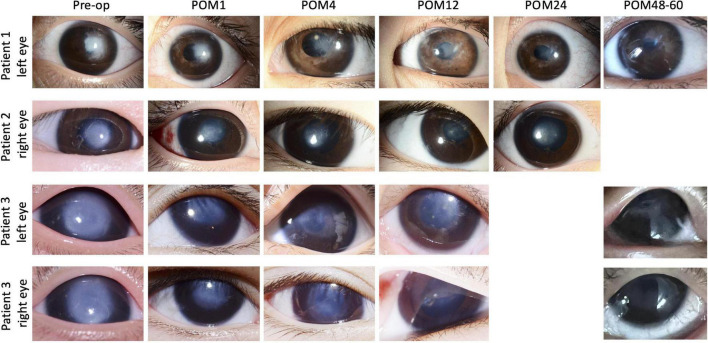
Picture collage of eyes which underwent selective endothelial removal for the treatment of Peters anomaly. The last reviews for patients 1 and 3 were at the 60th and 48th post-operative month, while the last review with photos being taken for patient 2 was at the 24th post-operative month. Gradual post-operative improvements in the central corneal opacities were seen in all eyes. Pre-op, pre-operative; POM, post-operative month.

This patient first presented to our clinic when she was 3 years old, with a visible left corneal opacity. A detailed clinical examination performed under sedation, in association with ocular ultrasound biomicroscopy (UBM), indicated the presence of multiple ocular anomalies including a dense central corneal stromal opacity, irido-corneal and lens-corneal adhesions, a secondary cataract and angle-closure glaucoma, thus leading to the diagnosis of Type 2 Peters anomaly. While lack of patient co-operation precluded accurate assessments of her visual acuity during all consultations, the presence of a persistent sensory exotropia of her left eye preceding her first visit indicated poor visual function and the presence of significant deprivation amblyopia. Her parents underwent counseling that any form of ocular surgery was primarily to improve ocular cosmesis, with little chance of visual recovery. At 39 months of age, this patient underwent crystalline lens aspiration, anterior vitrectomy, adhesiolysis, and SER for her left eye, with post-operative aphakia. She was discharged with a tailing course of topical steroid and antibiotic eyedrops which were eventually ceased. The most recent follow-up review for this patient was at the 60th post-operative month. While she remains uncooperative during assessments of her visual acuity, the central corneal opacity has cleared significantly in contrast to her pre-operative state, and it is now possible to clearly visualize details of the iris and pupillary margin through her cornea (Figure 2). She is currently steroid-independent, and only requires topical timolol eyedrops for treatment of her glaucoma.

### Case 2

The second patient was a 3 month-old infant, with a right eye corneal opacity which was noticed by her parents at birth. She was first reviewed by an ophthalmologist (not within our practice) at 3 months of age. Physical examination and UBM indicated the diagnosis of type 1 Peters anomaly, supported by the observed clinical features of a dense central corneal opacity (>5 mm diameter), iris-corneal adhesions, a normal crystalline lens and the absence of all other intraocular anomalies. Visual acuity of her right eye was estimated as 20/960 *via* forced preferential looking. She was immediately prescribed pharmacological mydriasis and occlusion therapy, which did not improve her visual function. She subsequently defaulted treatment up till 18 months of age, after which she was seen in our clinic for a second opinion. Her parents were counseled on the various surgical options available, and she finally underwent iridocorneal adhesiolysis combined with SER at 21 months of age. Thereafter, she received a tapering course of topical steroid and antibiotic eyedrops which were eventually ceased. Gradual post-operative clearance of the central corneal opacity (Figure 2) was accompanied by functional improvements in her visual acuity, which progressed from 20/94 at the 6th post-operative month to 20/30 at the 12th post-operative month. Visual acuity remained stable at 20/30, with a normal intraocular pressure of 15mmHg (Tono-Pen, Reichert Technologies, New York, NY, United States), during her most recent review at the 66th post-operative month. All post-operative visual acuities for this patient were estimated with the 3-m Kay Pictures test.

### Cases 3 and 4

**FIGURE 3:**
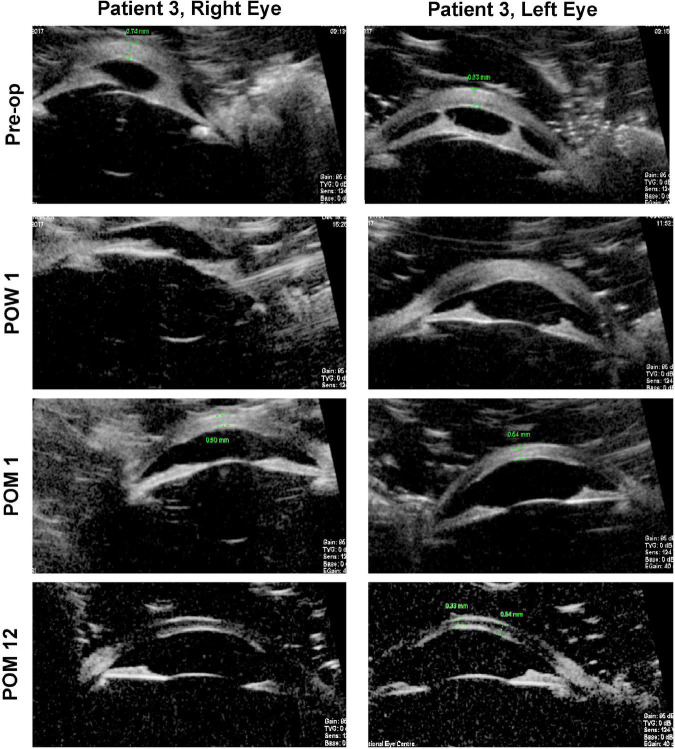
Ultrasound biomicroscopy (UBM) images for both eyes of patient 3. Pre-operatively, thick paracentral bands of iridocorneal adhesions with shallow anterior chambers may be seen in both eyes. Serial post-operative UBM images demonstrate that these bands have been lysed, with normalization of anterior chamber depths. Pre-op, pre-operative; POW, post-operative week; POM, post-operative month.

In contrast to the first two patients with unilateral disease, the third patient had bilateral type 1 Peters anomaly (Figure 2). Large central corneal opacities were noted at birth (>5.5 mm), and the patient was referred to our service at approximately 2 months of age. The observation of sensory nystagmus indicated bilaterally poor vision from early infancy. Baseline UBM revealed thickened irides with extensive bands of irido-corneal adhesions, as well as very shallow anterior chambers (Figure 3). This patient was prescribed pharmacological mydriasis from her first visit. However, this failed to improve her visual function or improve opacity. She was therefore scheduled for SER for her right eye at 9 months of age, followed by SER for her left eye. Serial post-operative UBM scans indicated successful lysis of all major bands of central iridocorneal adhesions associated with normalization of anterior chamber depths in both eyes (Figure 3). The bilateral central corneal opacities were also observed to have cleared gradually, with iris details now visible through both corneas. Her vision has improved from 20/470 in both eyes at the third post-operative month, estimated via the forced preferential looking technique, to 20/190 during her last review at the 48th post-operative month, estimated with the 3-m Kay Pictures test. Intraocular pressures were normal during the last review, at approximately 14mmHg (Tono-Pen, Reichert Technologies, United States) bilaterally. She is on no topical steroids.

## Discussions

**FIGURE 4:**
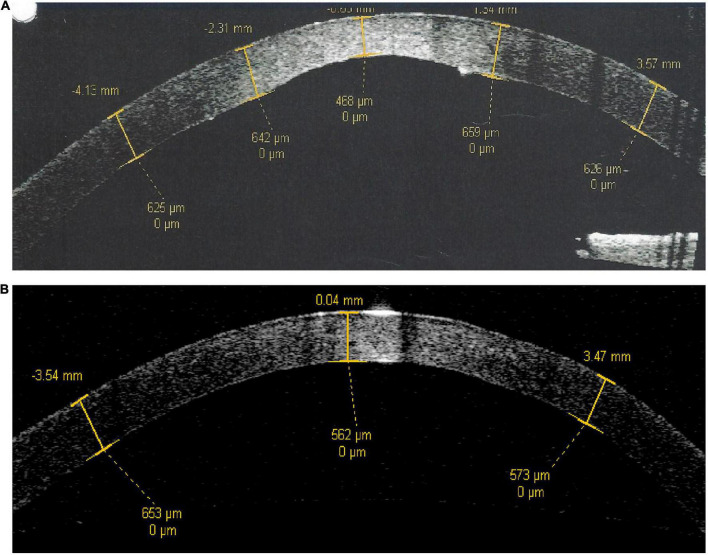
Anterior segment optical coherence tomography scans of patient 2. **(A)** Right eye with type 1 Peters anomaly. **(B)** Left eye which is normal. There is a posterior corneal defect in the central cornea of the right eye, evidenced by a central corneal thickness of 468 μm in contrast to a central corneal thickness of 562 μm in the normal fellow eye.

The cornea is a multi-layered tissue, with the epithelium and its basement membrane located most anteriorly, followed by the corneal stroma, terminating at the corneal endothelium and its basement membrane (also known as Descemet’s Membrane, “DM”) which are located most posteriorly (20). In the physiological state, corneal endothelial cells actively dehydrate the corneal stroma to maintain deturgescence, which is essential for stromal clarity. Excessive stromal hydration secondary to any corneal endothelial pathology leads to an opaque cornea (21). In Peters anomaly, there is focal attrition of corneal endothelial cells and/or DM in the central cornea. Corneal endothelial cells within the region of the paracentral irido-corneal and lenticulo-corneal adhesions surrounding this focal defect are anomalous as well. Anterior segment optical coherence tomography (ASOCT) clearly demonstrates a posteriorly excavated region within the central cornea in the right eye of patient 2, who has a diagnosis of type 1 Peters anomaly (Figure 4A, central corneal thickness 468 μm). This is in contrast to the appearance of a normal cornea such as in the fellow eye of the same patient (Figure 4B, central corneal thickness 562 μm). For Peters anomaly, pharmacologically-induced mydriasis to expand the visual axis and reduce the likelihood of amblyopia in the cases where the opacity is limited to the central cornea may result in good clinical outcomes (5, 22). When pharmacological treatments fail, surgical intervention should be considered as soon as medically possible (5). SER can be considered once the anterior chamber depth has developed to an adequate size for operation, approximately 6-months old, and before deprivation amblyopia develops.

The corneal endothelium comprises a monolayer of corneal endothelial cells. These cells secrete basement membrane material to form the DM, which supports corneal endothelial cell metabolism and adhesion. The DM comprises an anterior banded layer, and a posterior non-banded layer which gradually increases in thickness with age (23). As corneal endothelial cells are terminally differentiated and non-mitotic *in vivo*, the normal response to an endothelial defect would be enlargement and lateral migration of the surrounding endothelial cells to physically “fill” the defect, rather than extensive cellular division (24). We previously had performed *ex vivo* cell culture experiments on cadaveric human corneas which indicated that endothelial cells of young patients have the potential to migrate much faster than those of older patients (17). These concepts led us to the hypothesis that the lysis of irido-corneal and/or lenticulo-corneal adhesions (adhesiolysis), combined with selective removal of anomalous corneal endothelial cells within and around the region of the central corneal opacity in pediatric patients with Peters anomaly (i.e., SER), may subsequently induce centripetal migration of healthy peripheral corneal endothelial cells. SER thus has the theoretical potential to establish a functional monolayer over the focal dysfunctional endothelial layer (19). The successful repopulation of healthy endothelial cells over the central cornea contributes toward the restoration of corneal clarity *via* multifactorial pathways, such as by inducing clearance of anomalous glycosaminoglycans deposits (25), as well as reversal of corneal edema. Improvements in visual acuity are likely to continue for an extended period of time following SER for Peters anomaly, in a similar fashion to the gradual improvements in visual acuity observed over a 1–2 year period following endothelial keratoplasty in adult patients with severe corneal edema (26). Additionally, the importance of SER lies in the ability to use less-invasive management instead of corneal transplantation as a low-risk and viable alternative in certain cases. The most significant advantage of SER over pediatric corneal transplantation is its minimally invasive nature, the avoidance of allogenic tissue transplantation and the attendant complications associated with graft rejection, long-term immunosuppression and risk of graft infection in such young children (19, 27).

In this report, we have presented the long-term clinical outcomes of four eyes from three patients who have undergone SER for the management of Peters anomaly. There have been no post-operative complications directly associated with or attributable to SER amongst these four cases, up till the 48th post-operative month. The first patient we performed (case 2) is in fact at 66 months post-operatively, but due to travel restrictions from COVID-19, we have not seen the patient after the 36th-month follow-up. However, the patient’s local doctor reports that the patient is maintaining the same vision with a clear cornea. This is in contrast to the relatively high rates of complications such as graft rejection, secondary glaucoma, and cataracts which often occur as early as within the first 2 years following standard pediatric corneal transplantation (7, 11–13). While it has been generally difficult to measure the visual acuities of these pediatric patients due to other systemic factors, there appears to be a general trend indicating gradual improvements in vision and clearance of central corneal opacities for all patients in the post-operative phase (Figure 2). Additionally, all patients have avoided the need for long-term steroid medications as would be routine following standard pediatric corneal transplantation. While we have previously postulated that SER would be useful only for type 1 Peters anomaly (19), the favorable outcomes in Patient 1 suggest that SER may possibly be beneficial for selected cases of type 2 Peters anomaly as well.

While the long-term stability of post-operative outcomes associated with SER beyond a 48-month period is currently unknown, we suspect that the induction of corneal endothelial cell enlargement and redistribution by SER in early childhood may reduce the peripheral functional reserve of the corneal endothelium (28), leading to elevated risks of corneal endothelial decompensation in mid or late adulthood. However, we have also recently identified endothelial stem cell progenitors in the peripheral area of the corneal endothelium that can potentially replenish this peripheral reserve (29), which would theoretically mitigate corneal endothelial decompensation risks. Even if, in the worse case scenario, frank corneal endothelial decompensation was to necessitate corneal transplantation later in life, the clinical outcomes associated with corneal transplantation in an older pediatric population are known to be much more favorable than those associated with a younger population (30). These considerations support the view that SER may potentially be associated with a favorable long-term risk-benefit profile and provides a basis for further investigation of this surgical technique in a larger scale, phase II/III trial. Recently, published literature regarding the natural history of Peters anomaly, has indicated that up to 70% of these patients may experience spontaneous corneal clearance of the central visual axis without any treatment (31). However, these were most commonly found in eyes with normal irides (i.e., no adhesions), small corneal opacities, and the regression had occurred by a median age of 6.7 months. All our cases had iris abnormalities and presented at a later time frame. In addition, none of the patients with Type 2 Peters anomaly had spontaneous regression. In our case series, case 1 was diagnosed as Type 2 Peters anomaly. The authors also postulated that spontaneous clearance of the corneal opacity was most likely due to the centripetal migration of corneal endothelial cells from the peripheral cornea (31). In this context, we propose that SER may be viewed as a minimally invasive surgical procedure to accelerate the natural centripetal migration of healthy peripheral corneal endothelial cells/endothelial progenitor cells, in eyes with iris abnormalities, by removing the *in vivo* contact inhibition and iris adhesion.

Contact inhibition has been shown to be the leading cause of mitotic inhibition *in vivo*, and the removal of the contact inhibition between the endothelial cells by SER is the key stimulating factor for their migration (32). Hence, simple irido-corneal adhesiolysis alone at the adhesion point without SER over the whole corneal opacity area will not allow cellular migration and will not have effects on corneal opacity. Li et al. (33) also reported the restoration of corneal transparency after peeling off the keratolenticular adhesion and descemetorhexis around the region of demarcated corneal opacity in a Type 2 Peters anomaly patient. In addition, there has been a report describing the efficacy of primary descemetorhexis without graft placement (34), for the treatment of Type 1 Peters anomaly in an 8-year old child. It is encouraging that the visual acuity of the reported patient had improved from 6/36 to 6/20 within 12 weeks of the procedure (35). However, the abovementioned report (35) presented with significant thickening of the DM on ASOCT, rather than the DM attenuation which is seen in younger patients with Peters anomaly. In typical patients with Peters anomaly where a thickened DM is absent, the advantage of performing descemetorhexis rather than SER may be less obvious. In addition, performing a smooth descemetorhexis in such young children as in our cases is technically challenging due to adherence to the overlying posterior stroma and lack of corneal rigidity. Disturbance of the overlying stromal tissue can lead to posterior corneal nebulae. Another key advantage of performing SER over primary descemetorhexis is the fact that corneal endothelial cells are known to migrate very slowly across bare posterior corneal stroma, in contrast to over an intact DM, and can undergo endothelial-mesenchymal transition during this migratory process which can result in a loss of endothelial cell-specific markers and functional domains (17). As such, we feel that the DM should not be universally removed in all cases, and in fact should be retained whenever possible. Lastly, clinical studies have shown that topical administration of rho-associated protein kinase (ROCK) inhibitors accelerate the migration and proliferation of both healthy endothelial cells and transition-type of endothelial cells that surround the denuded area, in patients with Fuchs corneal dystrophy who underwent Descemet stripping only procedure (36, 37). However, for both commercially available and approved ROCK inhibitors, i.e., ripasudil hydrochloride hydrate ophthalmic solution 0.4% (GLANATEC; Kowa Co, Ltd., Nagoya, Japan) and netarsudil ophthalmic solution 0.02% (Rhopressa; Aerie Pharmaceuticals, Inc., Durham, NC, United States), the safety and effectiveness in pediatric patients below the age of 18 years have not been established (38, 39). In addition, we have shown that young donor age are factors that promote endothelial migration in an *ex vivo* human cornea culture model (17). As Peters anomaly occurs in infant population, we would expect the endothelial migration ability would be good, and ROCK inhibitors may not be required.

One of the limitations of this study is the small sample size. In addition, more cases are needed to confirm the beneficial effect of SER for Type 2 Peters anomaly. A comparative group with no intervention in one eye in bilateral cases may be considered in future studies to evaluate the tendency of spontaneous resolution of the corneal opacity.

In conclusion, SER for the treatment of Peters anomaly is associated with a favorable safety profile up to at least 48th post-operative month. This provides justification for the design and implementation of a larger phase II/III surgical trial to further verify the safety and to formally evaluate the efficacy of SER, beyond the initial four cases described in this report.

## Data availability statement

The raw data supporting the conclusions of this article will be made available by the authors, without undue reservation.

## Ethics statement

Ethical review and approval was not required for the study on human participants in accordance with the local legislation and institutional requirements. The patients/participants provided their written informed consent to participate in this study.

## Author contributions

Y-CL and YS collected the data, performed the analyses, and wrote the manuscript. VK and JM performed the surgery and supervised the manuscript writing. All authors contributed to the article and approved the submitted version.

## Supplementary material

Supplementary Video 1 Surgical video showing the surgical steps of Selective Endothelial Removal (SER).
